# Research Active Posterior Rhinomanometry Tomography Method for Nasal Breathing Determining Violations

**DOI:** 10.3390/s21248508

**Published:** 2021-12-20

**Authors:** Oleg G. Avrunin, Yana V. Nosova, Ibrahim Younouss Abdelhamid, Sergii V. Pavlov, Natalia O. Shushliapina, Natalia A. Bouhlal, Ainur Ormanbekova, Aigul Iskakova, Damian Harasim

**Affiliations:** 1 Department of Biomedical Engineering, Faculty of Electronic and Biomedical Engineering, National University of Radio Electronics, 61166 Kharkiv, Ukraine; yana.nosova@nure.ua (Y.V.N.); ibrahim.younouss.abdelhamid@nure.ua (I.Y.A.); 2 Department of Biomedical Engineering, Vinnytsia National Technical University, 21021 Vinnytsia, Ukraine; psv@vntu.edu.ua; 3 Department of Otorhinolaryngology, Stomatological Faculty, Kharkiv National Medical University, 61022 Kharkiv, Ukraine; shushliapina_nataliia775@ukr.net; 4 Azov Maritime Institute, National University “Odessa Maritime Academy”, 65000 Odessa, Ukraine; umkaser@web.de; 5 Faculty of Information Technology, Al-Farabi Kazakh National University, Al-Farabi Avenue 71, Almaty 050040, Kazakhstan; ain_25@mail.ru; 6 Institute of Automation and Information Technologies, Satbayev University, Satpaev Street 22, Almaty 050000, Kazakhstan; iskakova1979@mail.ru; 7 Faculty of Electrical Engineering and Computer Science, Institute of Electronic and Information Technologies, Lublin University of Technology, 20-618 Lublin, Poland

**Keywords:** nasal airways, human health, chronic respiratory disease, public health, CT, rhinomanometry

## Abstract

This study analyzes the existing methods for studying nasal breathing. The aspects of verifying the results of rhinomanometric diagnostics according to the data of spiral computed tomography are considered, and the methodological features of dynamic posterior active rhinomanometry and the main indicators of respiration are also analyzed. The possibilities of testing respiratory olfactory disorders are considered, the analysis of errors in rhinomanometric measurements is carried out. In the conclusions, practical recommendations are given that have been developed for the design and operation of tools for functional diagnostics of nasal breathing disorders. It is advisable, according to the data of dynamic rhinomanometry, to assess the functioning of the nasal valve by the shape of the air flow rate signals during forced breathing and the structures of the soft palate by the residual nasopharyngeal pressure drop. It is imperative to take into account not only the maximum coefficient of aerodynamic nose drag, but also the values of the pressure drop and air flow rate in the area of transition to the turbulent quadratic flow regime. From the point of view of the physiology of the nasal response, it is necessary to look at the dynamic change to the current mode, given the hour of the forced response, so that it will ensure the maximum possible acidity in the legend. When planning functional rhinosurgical operations, it is necessary to apply the calculation method using computed tomography, which makes it possible to predict the functional result of surgery.

## 1. Introduction

Modern medical diagnostics is based on an evidence-based approach, which is based on the use of high-precision equipment and methodologically correct information technologies to obtain reliable quantitative data regarding processes in the human body. Currently, the most active evolution is observed in functional diagnostic methods, which are aimed at registering quantitative indicators of the physiological functions of an organ, or the whole organism, and identifying their violations depending on a specific pathology [1,2]. This information is especially useful for a practicing physician−clinician, as it allows to link the anatomical, morphological and physiological parameters of the studied organ to clarify the picture of the pathological process. Functional research is also actively used in sports medicine, with professional selection and preventive examinations to determine the physical capabilities of the subjects [3].

### The Purpose of the Work

The aim of this work is to clarify the possibilities of posterior active rhinomanometry in the functional diagnosis of nasal breathing disorders and to clarify the correlation of functional data with structural changes in the configuration of the upper respiratory tract. It is also advisable to substantiate the possibilities of posterior active rhinomanometry in identifying respiratory–olfactory disorders and methodological features of rhinomanometric diagnostics.

## 2. Actuality of the Work

Due to the anatomical and physiological features and, accordingly, certain difficulties in testing nasal breathing, rhinology is one of the fields of medicine that is provided with the least amount of evidence for functional diagnostics [3,4,5,6,7]. This is manifested in the fact that, even taking into account the capabilities of modern rhinomanometric equipment and the corresponding specialized software, there is no clear correlation between the patient’s subjective sensations and the characteristics of the air flow during nasal breathing [8]. Increasing the accuracy of measurements and adjusting the indicators of the conditional age norm in the absence of a standard do not allow for solving this problem [9]. Research in the field of aerodynamics of the upper respiratory tract is the subject of the work of renowned specialists in the field of rhinology [10,11,12,13,14]. The latest trends are the study of the characteristics of the nasal cavity by CFD methods and the verification of simulation results with clinical data, as well as the prediction of functional results in rhino surgery [15,16,17,18]. The International Committee on Standardization of Rhinomanometry has been established and is currently functioning [19]. The results of studying the characteristics of the air flow in the nasal cavity, as well as the effect of the boundary layer on the mucous membrane of the nasal cavity, are the basic data for endoscopic surgery of the nasal sinuses [20,21]; planning of functional, minimally invasive surgical interventions [12,22,23], in particular septoplasty [22,23,24]; and functional−aesthetic rhinoplasty [25,26,27,28]. The influence of anatomical structures and changes in the architectonics of the nose on nasal aerodynamics [29,30,31,32,33,34,35] can be seen, in particular, in the syndrome of empty nose [30], odontogenic sinusitis [31], the shape of the nasal valve [32] and in sleep apnea syndrome [33,34,35], and the study of the characteristics of the air flow is carried out on full-scale models of the nasal cavity [36,37]. Moreover, studies of the olfactory function are relevant [38,39,40,41], especially in the diagnosis and subsequent rehabilitation of patients after COVID-19 [40]. The complexity of the problem, as well as various approaches and research methods, significantly complicate the interpretation of diagnostic data, which prevents the widespread introduction of equipment for testing nasal breathing in clinical practice, which leads to the use of rhinomanometry methods mainly for scientific research.

Therefore, currently relevant are the approaches aimed at improving the methods of functional diagnostics in rhinology by studying the effect of intranasal structures on the characteristics of the nasal air flow [12,29], ensuring the repeatability of data and substantiating additional diagnostic indicators, for example, in the study of the respiratory–olfactory function. It may also be advisable to modify the diagnostic equipment based on the analysis of the methodological features of testing nasal breathing, the study of nasal aerodynamics during forced nasal breathing [9] and the study of the aerodynamic parameters of the air flow in the nasal cavity under the action of odor vectors [41].

## 3. Analysis of Existing Methods of Nasal Breathing Research

Nasal breathing is the main, from the point of view of physiology, type of breathing, the disturbance of which can lead to a decrease in quality of life and serious complications, chronic hypoxia of the brain and other pathological conditions. Therefore, the study of aerodynamic nasal resistance, or its inverse value—the air conductivity of the nose—has always been one of the primary tasks in classical rhinology, ranging from the simplest tests to study the deviation of fluff by exhalation to the analysis of fogging exhalation phase time [9,12,15]. The considered methods, taking into account their quality and lack of evidence at the turn of the fourth industrial revolution, are certainly an anachronism because they are not based on quantitative criteria and intellectual analysis of instrumentally measured diagnostic results.

Currently, the most modern quantitative method for assessing the function of nasal breathing is rhinomanometry—a method in which measurements of the pressure drop in the nasal cavity and the corresponding flow of air passing through the nose [9,19,31,42,43,44]. The method of computer rhinomanometry generally accepted in the last two decades [9,19,42,43,44] allows for estimating the size of disturbance of nasal breath by definition of an indicator of aerodynamic nasal resistance in the form of a ratio of pressure difference in a nasal cavity to value of air flow volume. This value is respectively determined in kPa/(L/s) or in Pa/(cm^3^/s). Modern computerized rhinomanometers are complex electronic devices, which include miniature transducers of pressure and volume flow (or velocity) of air flow, which allow due to the original data processing for displaying graphical dependences of the parameters of air flow passing through the nasal cavity at breathing. Modern rhinomanometry methods are classified according to the measured indicators and the type of initial data processing. For example, rhinoresistometry is the most modern approach, in which aerodynamic nasal drag is calculated not on average, but directly at each point of the respiratory cycle.

**Figure 1:**
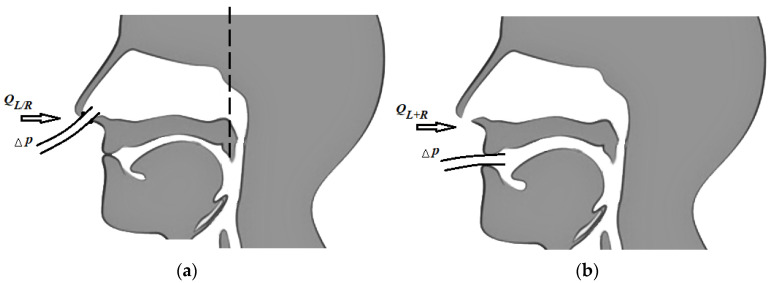
Illustration of measurements of pressure drop Δ*p* and air flow applying methods of active rhinomanometry: (**a**)—front, (**b**)—back (the dotted line shows the limit of measurement of pressure drop at front active rhinomanometry at the level of choanas).

The principles of rhinomanometry, based on the displacement of pressure measurement points, are classified into anterior and posterior [9,19,42]. During anterior active (natural respiration of the patient) rhinomanometry (see Figure 1a), a measuring tube is inserted in one of the nostrils, which is connected to the inlet of the pressure transducer to determine the pressure drop at the level of choanas and atmospheric and sealed obturator; therefore, this half of the nasal cavity is not involved in the process of respiration (the corresponding air flow *Q* is measured separately for each left and/or right half of the nose, respectively). The disadvantages of the method of anterior active rhinomanometry include inaccuracy of total nasal resistance, with forced breathing due to alternating obstruction of the nasal halves, the presence of a vasomotor reaction caused by the mucous membrane of the nasal cavity, which leads to a reflex change in the transverse dimensions of the obstructed nasal passage opposite the subject, as well as changes in the nasal mucovascular system between the nostrils.

The method of posterior active rhinomanometry involves the placement of a point for measuring the pressure in the nasopharynx (see Figure 1b) using a tube placed in the mouth with the lips tightly closed. The distal edge of the tube should not cause the studied vomiting reflex, which is especially important when examining pediatric patients. To perform this method, the patient may need to become accustomed to performing breathing maneuvers through training. This measures the total air flow *Q_L+R_* and the pressure difference Δ*p* between nasopharyngeal and atmospheric pressure, taking into account the area of the epipharynx. Despite certain methodological difficulties, this method actually allows the study of nasal breathing in the physiological conditions as close as possible to realism (real ones) and to test nasal breathing in (the) forced mode [3,9].

Regardless of the location of pressure measurement points and measurement methods, rhinomanometry provides for mandatory measurements and analysis of two indicators: the pressure drop and the air flow during nasal breathing. Methods involving the analysis of only one of the parameters, for example, only air flow, are essentially called rhinoflowmetry, nasal spirometry or pneumotachometry [3,45]. They allow only to compare air flow, for example, during nasal and oral respiration and do not allow to estimate the value of the coefficient of aerodynamic nasal drag. Alternatives are the devices that allow to determine the dynamic pressure in the nasal cavity by inserting a measuring tube into the appropriate sections of the nasal passages to determine the dynamic pressure and establish the relative flow of air through the nasal passages.

Many publications have been devoted to the method of anterior active rhinomanometry, its hardware implementation, the analysis of indicators and the algorithms for processing the resulting data [19,42,43,44]. Therefore, it is expedient to pay attention to the method of posterior active rhinomanometry, justification of additional indicators of nasal breathing, methodological errors and correlations between functional data and the architecture of the nasal cavity according to computed tomography.

The method by which it is possible to study the configuration of the structures of the nasal cavity quite comprehensively is X-ray computed tomography [12,13,14,31,46,47,48,49,50,51,52]. The method allows for visualizing both bone structures and soft tissues and to segment the airways [7,12,31] by digital tomographic image processing [31,49,50,51,52]. In addition, CFD models, which are based on computed tomography data, allow to study the aerodynamic characteristics of the upper respiratory tract [7,10,13,23,53,54,55,56,57,58,59,60]. The works in which the correlation of the results between the data of computed tomography, rhinomanometry and acoustic rhinometry are studied are quite relevant [22,61,62,63].

## 4. Materials and Methods

To perform anterior active rhinomanometry (AARM), a developed and patented computer rhinomanometer (see Figure 1) for testing nasal breathing TNDA with a block of differential consumption characteristics of PVC (certificate of state metrological certification in Ukraine, № 05-0102 of 01.04.2010 d.) [3,9] is used, which implements the principle of posterior active rhinomanometry (PARM). The main advantages of the method, despite the more common anterior active rhinomanometry, in the opinion of the authors, are the ability to breathe through both halves of the nose and, consequently, greater physiological study and the ability to test for forced breathing, where most nasal breathing difficulties occur. The original software has been developed for the device, which allows to observe breathing patterns in real time, receive data from sensors, calculate additional parameters in spreadsheet format and calibrate the device, taking into account the ambient temperature and atmospheric pressure.

**Figure 2:**
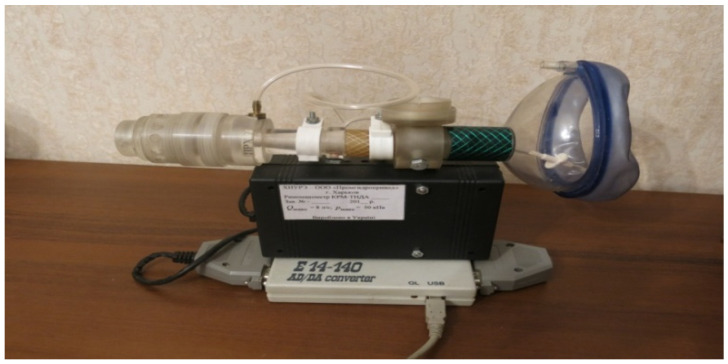
Computer rhinomanometer TNDA.

The device presented in Figure 2 includes a measuring unit containing pressure and air flow sensors, the signals from which are transmitted to the conversion unit, in which the output signals of the sensors are digitized using an ADC and transmitted through the interface module to the PC. The control microcontroller generates the necessary strobe pulses according to the selected mode. High-level signal processing, visualization, analysis and logging of examination results are performed on a PC using software.

**Figure 3:**
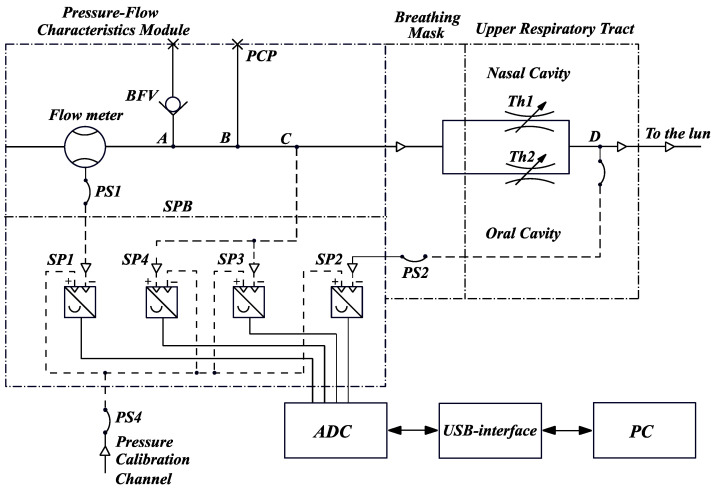
Combined scheme of a computer rhinomanometer TNDA (PSP—pressure control point, BFV—check valve adapter, Th1 and Th2—parallel adjustable throttles (resistances), SP1-SP4 pressure transducers, PS1–PS4—flexible hoses).

The combined scheme of a computer rhinomanometer TNDA (Figure 3) consists of a unit for determining the differential flow characteristics (designation according to the certificate of metrological attestation № 05-0102 of 01.04.2010) and an elastic mask with pipelines, imposed tightly on the face (nose and mouth area) of the patient. The rhinomanometer used by us consists of a flow meter, which is installed in the air path, a block of pressure transducers SPB, an analog-to-digital converter ADC module, a USB interface and a computer PC.

An internal cylindrical diffuser with expansion towards the source of air consumption is located in the flowmeter body based on the Venturi nozzle. An adapter with a BFV check valve and PSP pressure control point is attached to the flowmeter body. A mask with an inlet channel C for the passage of inhaled and exhaled air and channel D in the form of a flexible sleeve PS2 entering the inside of the mask and serving to communicate with the patient’s oral cavity by holding the tip of the sleeve with his lips is mounted to the outlet of the adapter (it is advisable to use a rigid plastic mouthpiece to exclude the possibility of clamping the lips or teeth of the flexible sleeve).

The patient’s nasal passages are schematically shown as parallel adjustable throttles (resistances) Th1 and Th2, each of which includes the resistances of the valve (wings of the nose) and the nasal passage proper. Adjustability of throttles means the variability of the cross-sectional values of the nasal passages depending on the specific condition of the patient, for example, before and after rhinoplasty when correcting the curvature of the nasal septum.

The SPB unit contains SP1, SP2, SP3 and SP4 pressure transducers with electrical connectors and flexible hoses for connecting pressure transducers with a flowmeter (PS1) and D oral cavity (PS2 and PS3). Flexible hose PS4 is used for simultaneous testing (during verification) of pressure transducers SP1, SP2, SP3 and SP4. The pressure is measured by appropriate sensors at the following points:

SP1—pressure (vacuum) in the flow meter;

SP2—pressure (vacuum) in the patient’s oral cavity (at point D) behind the nasal passages (throttles Th1 and Th2);

SP3—pressure (vacuum) at the mask inlet (channel C);

SP4—overpressure at the mask outlet (channel C).

The BFV check valve, connected at point A, serves to limit the overpressure during exhalation to avoid damage to the SP1, SP2, SP3 and SP4 transducers and to prevent the mask from coming off the patient’s face due to the high aerodynamic resistance of the Venturi nozzle. The PCP pressure test point B is used to connect an optional pressure transducer if required.

Thus, when testing the patient, the air flow rate inhaled through the nose and the pressure drop across the resistances Th1 and Th2 are determined simultaneously on two nasal passages, or alternately, measurements are taken in the inspiratory phase. The processing of test results is carried out by plotting the graphical dependence of the pressure drop on the flow rate and calculating the ratio of the pressure drop to the flow rate and air flow power. When the air flow is reversed (expiratory), only the excess pressure is monitored, which is measured with the SP4 transducer to indicate the expiratory phase. When using a check valve, the SP4 sensor does not exceed 100 Pa.

The device is characterized by the following measured values:

- The maximum pressure drop across the nasal cavity during forced inhalation can reach values up to 50 kPa;
- The maximum air consumption during breathing in the inhalation cycle is 8 L/s;
- ADC capacity—12 bits (4096 levels), while the quantization step significantly exceeds the error of 0.5% of the maximum signal value, which is accepted for medical equipment;
- The sampling rate of data from the measuring converters on the ADC is 500 Hz, which makes it possible to effectively study the breathing pattern with high-frequency components not exceeding several tens of Hz;
- The device is characterized by the simplicity of performing preparatory procedures by medical personnel (sterilization, adjustment, calibration) and, directly, the examination itself (selection of measurement modes, data analysis and visualization).

The methodology for examining patients using a computer rhinomanometer TNDA to obtain data on inspiratory dynamic posterior active rhinomanometry with forced breathing is as follows:

- Immediately before the examination, data of atmospheric pressure and air temperature are entered, which affect the accuracy of the measurement results;
- The patient puts on the mask and fixes its maximum fit by creating a vacuum during inhalation with a closed air inlet of the Venturi flow meter;
- The mouthpiece of the differential pressure transducer is placed in the oral cavity by 2–3 cm at a slight upward angle, so that there is no gag reflex, and the distal opening of the mouthpiece is not blocked by the structures of the tongue, hard palate or saliva; at the same time the lips are tightly compressed, preventing breathing through the mouth;
- The mode of recording the examination data is set, and the patient performs 10 maximally forced breathing cycles through his nose;
- The results of the survey (data from the respiratory cycle, peak and averaged over several breathing cycles air flow rates and pressure drops) are entered into the survey file.

For tomographic studies, a SOMATOM + spiral X-ray tomograph of SIEMENS firm (Munich, Germany) was used. For processing, we used tomographic sections in the axial projection parallel to the orbital–meatal line with a step of 1 mm and a spatial resolution of 0.4 mm. The initial data for the conducted research are sets of images of tomographic sections.. The images are stored in DICOM format [12,31,46,47,48,49,50], converted using the standard utility DICOM_IMAGE in raster format BMP (Windows bitmap) with a size of 512 × 512 (x × y) and eight-bit representation of intensity levels (after window-range for soft tissue preprocessing). Preliminary processing of tomographic images was performed by the method of median filtration [12,31,49,50] to eliminate possible interference in the form of pulsed noise. Methods of digital filtering, image segmentation and analysis, fluid dynamics, signal processing and statistical analysis are used [3,9,12,31,49,50,51,52,53].

Our study considers 346 cases. We examined adult patients aged 22 to 65 years. All patients were divided into groups: control group—60 patients (30 males and 30 females), acute chronic rhinosinusitis—76 (40 males and 36 females), curvature of the nasal septum—132 (65 males and 67 females), adenoid vegetation—14 patients (9 males and 5 females); additionally, there were 64 patients (31 males and 33 females) with respiratory impairment of smell, and among them, after COVID-19, there were 28 patients (14 males and 14 females). The potential of the upper respiratory tract and individual physiological variability were assessed by us using inspiratory spirometry. Rhinomanometry and tomographic preliminaries were carried out on the basis of the Kharkiv Regional Clinical Hospital, Center for Emergency Medical Assistance and Disaster Medicine in the Neck, Head and Clinical Surgery Department.

## 5. Methodological Features of Dynamic Posterior Active Rhinomanometry and Main Indicators of Nasal Dysfunction

The device with which the signals were recorded belongs to the experimental production of NURE (see Figure 2).

**Figure 4:**
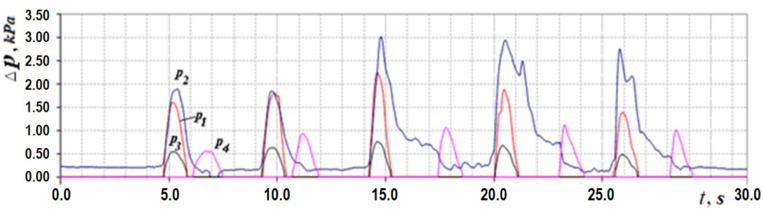
Respiratory cycle diagram according to dynamic PARM data.

Let us consider the features of posterior active rhinomanometry and cyclograms of nasal breathing. Typical cyclograms of nasal breathing during posterior active rhinomanometry are shown in Figure 4. It should be borne in mind that measurements according to the rhinomanometer diagram shown in Figure 2 are carried out in the inspiratory phase, as evidenced by a zero reading of the p4 transducer and a nonzero value of the pressure on the p1 transducer, set in a flow sensor of the Venturi nozzle type. The pressure readings on the SP1, SP2, SP3 and SP4 pressure transducers will correspond further in the text and in the diagrams to the values p1… p4 ($p_{1}\leftarrow SP1;\;p_{2}\leftarrow SP2;\;p_{3}\leftarrow SP3;\;p_{4}\leftarrow SP4$).

The PARM method provides for measuring the total air flow rate *Q* when breathing through the nose through both nasal passages and the pressure difference Δ*p* between atmospheric and in the nasopharynx (the distal end of the measuring tube of the *p*_2_ pressure transducer is introduced through the oral cavity). Thus, according to the diagram shown in Figure 3, the determination of the air flow rate can be carried out using the pressure transducers located in the diffuser (according to the principle of operation of the Venturi nozzle), the pressure transducer *p*_1_ and the pressure drop Δ*p* across the nasal passages, which is determined as the difference between the values of the differential pressure transducers *p*_2_ and *p*_3_:

(1) $$\Delta p=p_{2}-p_{3},$$

taking measurements in the oral cavity and at the entrance to the nasal passages (inside the mask). The transducers *p*_1_, *p*_2_ and *p*_3_ measure the vacuum in relation to atmospheric pressure (in the inspiratory cycle), and the transducer *p*_4_ is overpressure in the expiratory cycle to fix the respiratory phases, which can be seen in Figure 4. Considering that the pressure transducers are differential, it can assume that the values are equivalent $\Delta p_{*}=p_{*}$, where the index ( ) denotes any of the sensors used. The main diagnostic indicators, in addition to directly measured values of air flow *Q_H_* and pressure drop Δ*p*, are:

*A*—new aerodynamic coefficient of resistance, according to the formula:

(2) $$A=\frac{\Delta p}{Q},$$

*N*—breathing power, according to the formula

(3) N=Δp⋅Q

**Figure 5:**
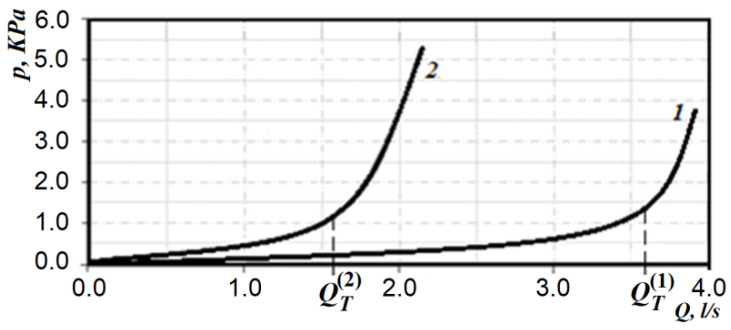
Typical rhinomanometric dependences of pressure difference on air flow in norm (1) and at disturbance of nasal breath (2).

Typical rhinomanometric dependences of the pressure drop on air flow in forced breathing are normal and in case of nasal breathing disorders are shown in Figure 5.

It is obvious from Figure 5 that the maximum air flow at the conditional rate is significantly (almost two times) greater than with a persistent violation of the new breath. The pressure drops do not differ much, but it can be seen that in cases of nasal breathing disorders, there is increasing air flow due to the disproportionate increase in pressure drop. This can be seen from the pneumatic breathing power (according to Formula (3)), which is 0.14 W in normal and 0.11 W in nasal breathing disorders, but with increased pressure drop and significantly reduced air flow, respectively. The increase in the pressure drop in nasal breathing disorders is associated with the patient’s attempt to create a greater pressure gradient due to the strong tension of the respiratory muscles and due to it to developing increased air flow. However, the graphs in Figure 5 show that the relatively rapid transition to the area, which is expressed by the quadratic dependence of the pressure drop on air flow, leads to the fact that the amount of air flow increases quite slowly and even when approaching the norm causes excessive rapid fatigue and, as a consequence, the transition to non-physiological oral respiration. In fact, both in the norm and in nasal breathing disorders, the dependence of the pressure drop Δ*p* on air flow *Q* has two areas: linear (in the area of relatively low costs), and nonlinear–quadratic (in the area of high costs). In fact, this dependence of the pressure drop on the flow of air in the nasal cavity during respiration can be represented as follows:

(4) $$\Delta p=\left\{ \begin{array}{l} f(Q);\;if\;Q<Q_{T};\\ f(Q^{2});\;if\;Q\geq Q_{T}, \end{array}\right.;$$

where $Q_{T}$ is the amount of air flow during the transition to quadratic mode.

In this case (without considering separately the region of the transient mode of air flow), it is possible to form a criterion according to the definition of the transition point from the region of linear dependence of pressure drop from air flow to quadratic—which is characterized by turbulent (quadratic) air flow. The higher the flow point, the more potential it has to achieve airflow during nasal breathing. The corresponding transition points $Q_{T}^{\left(1\right)}$ and $Q_{T}^{\left(2\right)}$ under different states are shown in Figure 5.

Thus, it is possible to modify the method of data analysis of forced posterior active rhinomanometry by introducing a criterion for the effectiveness of nasal breathing, which includes determining the transition point *Q_T_* from the laminar linear mode of air flow to turbulent quadratic according to the following formula:

(5) $$k_{T}=\left(1-\frac{Q_{\max}-Q_{T}}{Q_{\max}}\right)\cdot 100\%;$$

where *Q*_max_—maximum air flow, *Q_T_*—threshold value of air flow during the transition to the quadratic flow regime.

The range of values *k_T_*, which is determined by the Formula (5), can be divided into sub-bands that characterize the low, medium and high energy efficiency of forced nasal breathing. This indicator is based on data from our own research—statistical processing of nasal breathing test results in 245 patients (see Table 1). At the same time, the indicators of maximum pressure drops and air flow during forced breathing and fatigue and endurance of patients during respiratory tests were taken into account.

Determination of the transition point from the laminar mode of air flow to turbulent quadratic is based on the numerical differentiation $\frac{dp_{i}}{dQ_{i}}$ of the original data, which are given as a discrete function $p_{i}=f(Q_{i})$, and the fulfillment of the 10% empirical condition $\frac{dp_{i}}{dQ_{i}}\geq 0,1\cdot {\left(\frac{dp_{i}}{dQ_{i}}\right)}_{\max}$.

The next indicator that characterizes the individual physiological variability and capabilities of the lungs and lower respiratory tract is the efficiency of the aerodynamic conductivity of the nose, which is defined as:

(6) $$k_{Q}=\frac{Q_{N}}{Q_{M}}$$

where *Q_n_* and *Q_m_* are the volumetric flow of air when breathing through the nose and mouth, respectively. Hereinafter in the notation, unless otherwise indicated, the volume flow rate is the volume flow rate through nasal breathing *Q_N_* ≡ *Q*.

It is also advisable to use an indicator of the efficiency of the nasal valve. The main function of the nasal valve is its ability to limit the flow of air through dynamic throttling. This effect is achieved due to the mobility of the external anatomical structures of the nasal valve, and it can be determined by the ratio of the number of respiratory cycles with limited flow when breathing through the nose *n*(*Q*) to the total number of respiratory cycles *n*_Σ_(*Q*):

(7) $$k_{NV}=\frac{n(Q)}{n_{\Sigma }(Q)}\cdot 100\%.$$

**Figure 6:**
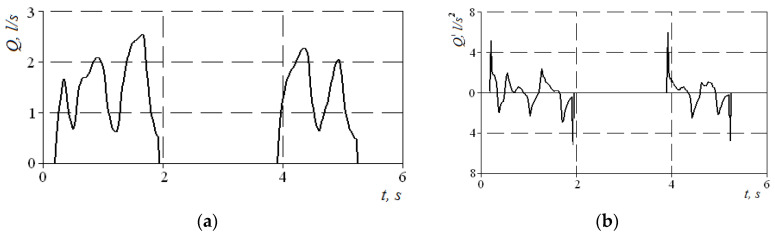
Graphs of the air flow signal in two respiratory cycles when inhaled according to the forced dynamic PARM (**a**) and derived from the air flow time (**b**).

This indicator can be determined visually by the cyclogram of respiration (see Figure 4), where the first three cycles have pointed peaks, and the last two are flattened with obvious signs of air speed limitation. Normally, the signal of air flow in the respiratory cycles has a periodic form with pronounced maxima. Moreover, when forced breathing under the action of discharge inside the nasal cavity occurs, the movable wings of the nose—the external anatomical structures of the nasal valve—forming the lateral walls of the nasal cavity at the entrance to the nasal passages are shifted in the medial direction and reduce the area of living section aerodynamic drag (in the extreme case to complete obstruction of the nasal passage), preventing an increase in air flow and creating local extremes. Therefore, in the automated mode, such features can be established according to the numerical differentiation of the air flow cyclogram (see Figure 6a) and the determination of a large number of extrema on the cyclogram by changing the sign of the derivative (see Figure 6b). The main features here are that the testing must be performed only in forced breathing mode and the function of the nasal valve is considered satisfactory in at least 50% of cycles with limited air flow. Thus, it is possible to determine a quantitative criterion for assessing the operation of the nasal valve to regulate air flow.

**Figure 7:**
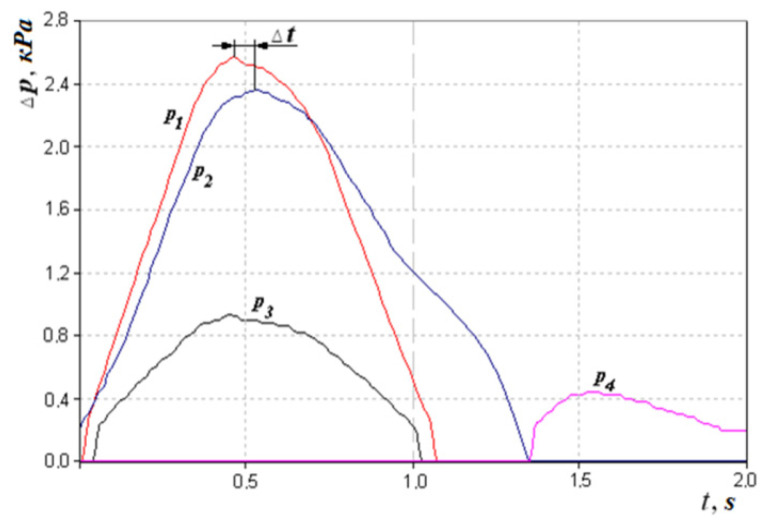
Respiratory cycle diagram showing the time shift Δ*t* between the amplitudes of the pressure transducer signals *p*_1_ and *p*_2_.

The next indicator can be considered the loss of friction of air on the mucous membrane of the nasal cavity as shown in Figure 7. This can be indirectly defined as the phase difference between the signals of pressure drop and air flow,

(8) Δφ=φ(Δp)−φ(Q),

or taking into account the method and scheme of measurement (Figure 3 and Figure 7).

Regarding the example of the respiration cyclogram (see Figure 7) and given that Δ*ϕ* and Δ*t*, we can determine that the time shift Δ*t* between the maxima of the pressure difference *p*_2_ signals in the nasal cavity and the pressure *p*_1_ in the flow meter is 0.05 s, which corresponds to the phase shift between signals *δ* = 9°. However, determining the statistical significance of this indicator in the diagnosis of diseases of the upper respiratory tract requires further study and medical justification depending on, for example, the condition and humidity of the mucous membrane of the nasal cavity.

Also of interest is the magnitude of the surplus vise in the presence of the pressure (the difference between the choanal and nasopharyngeal vise).

**Figure 8:**
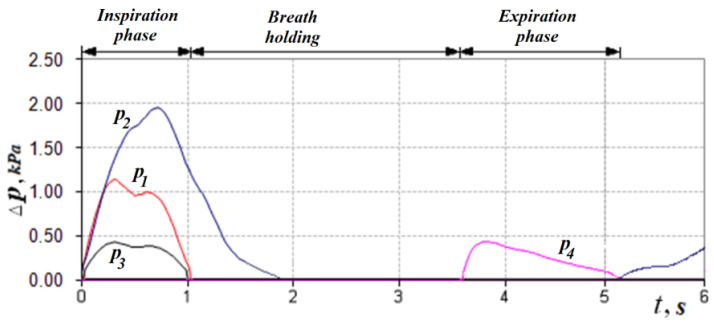
Respiratory cycle diagram according to dynamic RAP data when communicating with the oral cavity from the nasopharynx in the phase of holding the breath.

**Figure 9:**
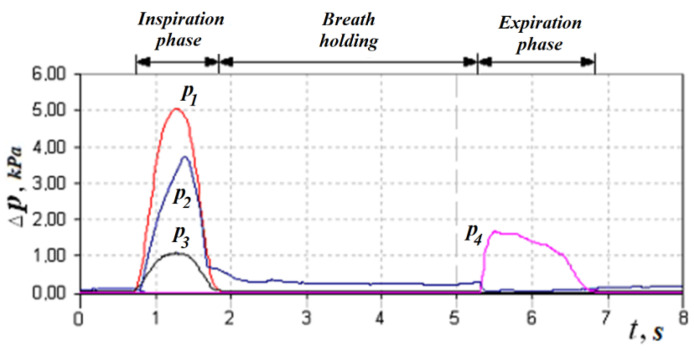
Respiratory cycle diagrams according to dynamic AAPM data with a hermetic separation of the oral cavity from the nasopharynx by the structures of the soft palate in the phase of holding the breath.

In accordance with the above design features, in the inhalation phase (see Figure 4, Figure 8 and Figure 9), which is fixed by the non-zero value of the pressure transducer p_1_ installed in the flow meter based on the Venturi nozzle, the pressure signals of the transducers *p*_1_, *p*_2_ and *p*_3_, and that record the vacuum, reach their maximum value; while holding the breath, the signals of all transducers are equal to zero. The expiratory phase is recorded by non-zero readings of the pressure transducer.

**Figure 10:**
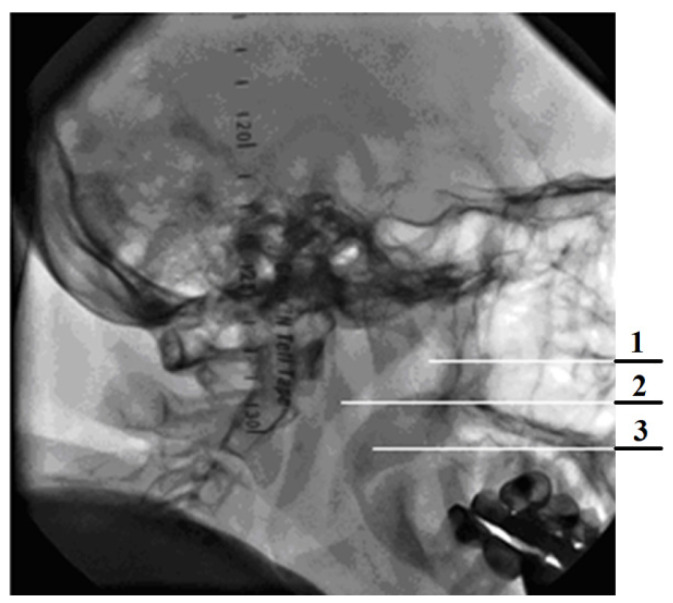
Static image of a frame of high-speed dynamic radiography of the nasopharynx in a sagittal projection (1—airway of the nasopharynx, 2—posterior pharyngeal wall, 3—soft palate).

The readings of the transducer p_2_ that measures the pressure in the nasopharynx (at the exit from the channel), the distal end of the measuring tube of which is located in the oral cavity, can be nonzero if the oral cavity is sealed from the nasopharynx by the structures of the soft palate (see Figure 8 and Figure 9) during breath holding and can be about 100 Pa. Anatomical and physiological explanations for this phenomenon are shown in Figure 10. This indicator can be of diagnostic value when studying the degree of mobility of the soft palate, for example, in the treatment of snoring and the syndrome of obstructive sleep apnea [35].

## 6. Aspects of Verification of Rhinomanometric Diagnostics Results According to Spiral Computed Tomography Data

Rhinomanometry indicators present the magnitude of nasal breathing disorders relative to the conditional physiological and age norms but do not indicate the cause and location of structures that affect the characteristics of air flow. Therefore, it is advisable to use the method of introscopic imaging, which would reflect the anatomical configuration of the nasal cavity, such as spiral computed tomography, and consider the influence of nasal canal architecture on nasal aerodynamics in typical pathological changes. Furthermore, according to tomographic data and with the help of analytical and CFD methods of flow calculation, it is possible not only to visually assess the configuration violation but also to determine the aerodynamic drag of the nasal cavity at the appropriate cross-sections perpendicular to the air flow. For this purpose, it is necessary to receive the aerodynamic model of a nasal cavity on the basis of segmented sections of airways.

**Figure 11:**
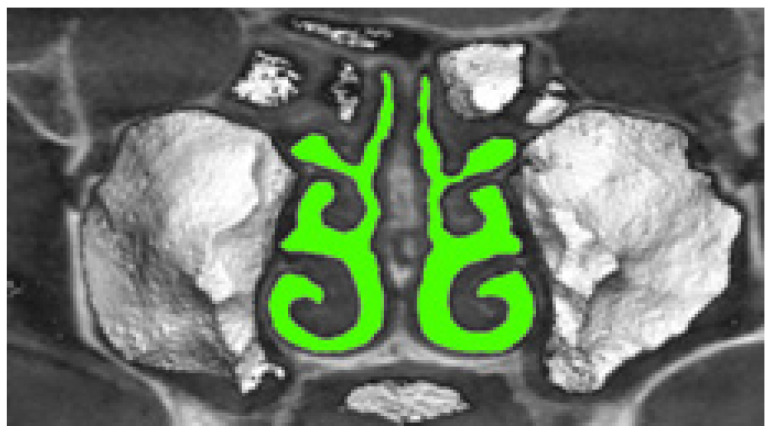
Illustration of airway segmentation by a three-dimensional voxel model of the nasal cavity.

The airway segmentation of the three-dimensional voxel model of the nasal cavity, obtained from tomographic data, is illustrated in Figure 11 and is performed at the intensity $T_{HU_{A}}$ threshold for air density according to the formula (in the soft-tissue window mode we used, the air density threshold was chosen to be less than 0):

(9) $$c(x,y,z)=\left\{ \begin{array}{l} 1;\;if\;b(x,y,z)\leq T_{HU_{A}};\\ 0;\;if\;b(x,y,z)>T_{HU_{A}}{}_{T}, \end{array}\right.,$$

where b(x,y,z) is the original halftone model of the upper respiratory tract, and c(x,y,z) is the binary voxel model of segmented airways of the nasal cavity.

Airway sections are obtained on the basis of multiplanar reconstructions of tomographic data in the frontal plane. The aerodynamic model takes into account the conditions of flow continuity and constancy of the pressure drop in the nasopharynx:

(10) $$Q=Q_{L}+Q_{R}$$

(11) $$\Delta p=\Delta p_{L}=\Delta p_{R}$$

where *Q_L_*, *Q_R_* and *Q* are the air flow through the left and right nasal passages and the total, respectively, and Δ*_p_*, Δ*_pL_* and Δ*_pR_* are the pressure drops during respiration through the left and right nasal passages and total, respectively. Given that during forced breathing according to Figure 5 and Formula (4), the air flow regime will be turbulent (square), according to conditions (10) and (11) the pressure drops in the nasal cavity will be defined as:

(12) $$\Delta p=\Delta p_{L}=\Delta p_{R}=Q_{L}^{2}\cdot A_{L}=Q_{R}^{2}\cdot A_{R}$$

and represent the sum of the pressure loss along the length ($\sum \Delta p_{l_{L}}$ and $\sum \Delta p_{l_{R}}$) of the nasal passage of each half of the nasal cavity and the local resistance ($\sum \Delta p_{r_{L}}$ and $\sum \Delta p_{r_{R}}$) at the cross sections:

(13) $$\Delta p_{L}{}^{}=\sum \Delta p_{l_{L}}+\sum \Delta p_{r_{L}}=Q_{L}^{2}A_{L},$$

(14) $$\Delta p_{R}=\sum \Delta p_{l_{R}}+\sum \Delta p_{r_{R}}=Q_{R}^{2}A_{R},$$

Based on the small length of the nasal cavity (about 70 mm) and the mutual influence of local resistances, which are located at a short distance, their amount should be replaced by the maximum of them. Then, according to [12,31,64], the common supports along the left and right channels of the nasal cavity of Formulas (13) and (14) can be represented as:

(15) $$A_{L}=\sum_{}^{}\lambda_{L}\cdot \rho \frac{L_{L}}{d_{h_{L}}\cdot 2S_{L}^{2}}+\max\left(\xi_{L}\cdot \rho \frac{1}{2S_{L}^{2}}\right),$$

(16) $$A_{R}=\sum_{}^{}\lambda_{R}\cdot \rho \frac{L_{R}}{d_{h_{R}}\cdot 2S_{R}^{2}}+\max\left(\xi_{R}\cdot \rho \frac{1}{2S_{R}^{2}}\right),$$

where $\lambda_{L}$, $\lambda_{R}$—coefficients of pressure loss in length for the left and right nasal passages, respectively;

$\xi_{L}$, $\xi_{R}$—coefficients of local resistance of the left and right nasal passages, respectively;

$L_{L}$, $L_{R}$—lengths of sections for the left and right nasal passages, respectively;

$Q_{L}$, $Q_{R}$—air flow through the left and right nasal passages, respectively;

$S_{L}$, $S_{R}$—cross-sectional areas of the left and right nasal passages, respectively;

ρ—air density;

$d_{h_{L}}$, $d_{h_{R}}$—equivalent cross-sectional diameters for the left and right nasal passages, respectively, which, taking into account the complex configuration of the nasal cavity [64], are determined by the formula:

(17) $$d_{h}=\frac{4S}{P},$$

where *S* and *P*—the area and perimeter of the section, which are determined numerically according to the segmented binary promissory note model of the nasal cavity. When determining local aerodynamic drag, their types are determined; for example, sharp expansion or contraction, throttle washer, latch and flow rotation, as well as their parameters, are measured [12,31]. The resulting coefficient of aerodynamic drag is defined as the equivalent aerodynamic drag of parallel channels in turbulent mode and is calculated according to expressions (10) and (12) of formula:

(18) $$Q=Q_{L}+Q_{R}=\sqrt{\frac{\Delta p}{A_{L}}}+\sqrt{\frac{\Delta p}{A_{R}}}=\sqrt{\frac{\Delta p}{A_{}}},$$

where:

(19) $$A={\left(\frac{\sqrt{A_{L}}\cdot \sqrt{A_{R}}}{\sqrt{A_{L}}+\sqrt{A_{R}}}\right)}^{2}.$$

**Figure 12:**
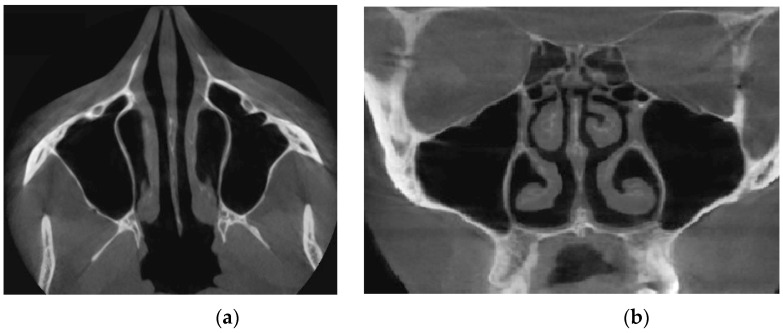
Computed tomography of the patient at the conditional norm: (**a**) axial tomographic section; (**b**) multiplanar reconstruction in frontal projection.

Verification of rhinomanometric data should be performed on the basis of tomographic data of the conditional norm and pathological conditions, which, due to various factors, affect nasal aerodynamics in different ways. Thus, in Figure 12 it is possible to see the characteristic tomographic sections of the nasal cavity at the conditional norm in the axial (Figure 12a) and frontal (Figure 12b) planes.

**Figure 13:**
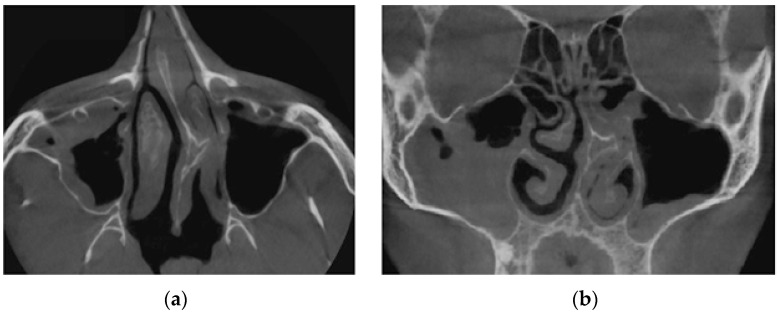
Computed tomography of a patient with curvature of the nasal septum to the left in the middle section: (**a**) axial tomographic section; (**b**) multiplanar reconstruction in frontal projection.

Figure 13 shows tomographic data of a patient with a curvature of the nasal septum to the left in the middle section, where you can clearly see a large change in configuration in the middle section of the nasal cavity on both axial (Figure 13a) and frontal (Figure 13b) sections.

**Figure 14:**
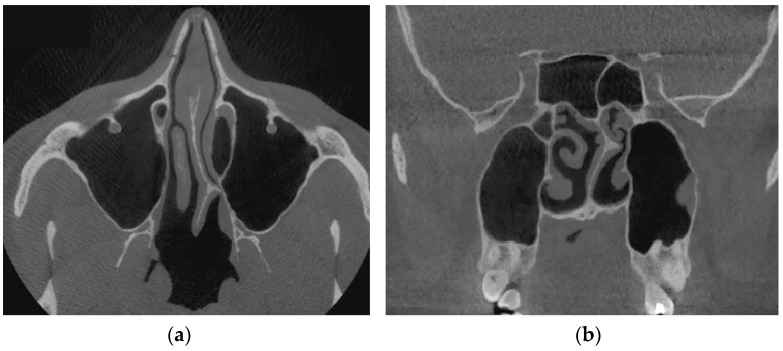
Computed tomography of a patient with curvature of the nasal septum to the left in the posterior part: (**a**) axial tomographic section; (**b**) multiplanar reconstruction in frontal projection.

Figure 14 provides a visualization of the local curvature of the nasal septum to the left in the posterior part of the nasal cavity, which is displayed on the axial—see Figure 14, and the frontal—Figure 14b tomographic sections.

**Figure 15:**
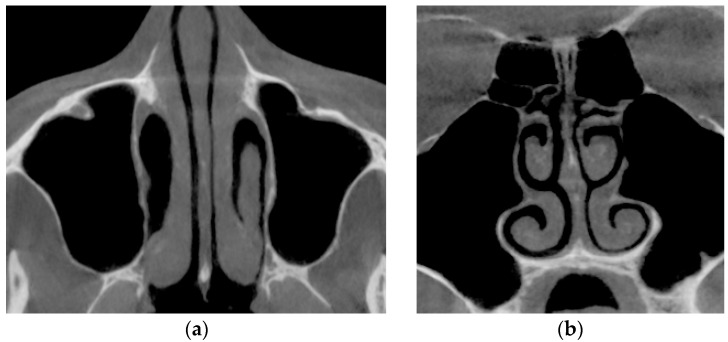
Computed tomography of a patient with the consequences of chronic rhinosinusitis: (**a**) axial tomographic section; (**b**) multiplanar reconstruction in frontal projection.

Figure 15 shows the thickening of the nasal mucosa in chronic rhinosinusitis, which leads to narrowing of the airways (see Figure 15a,b).

**Figure 16:**
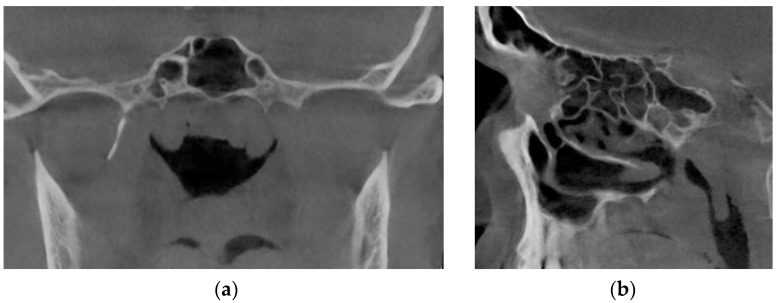
Computed tomography of a patient with adenoid vegetation: (**a**) axial tomographic section; (**b**) multiplanar reconstruction in sagittal projection.

Figure 16 demonstrates the state of the nasal cavity with adenoid vegetations, which can be seen in the images of characteristic sections in axial (Figure 16a) and sagittal (Figure 16b) projections.

**Figure 17:**
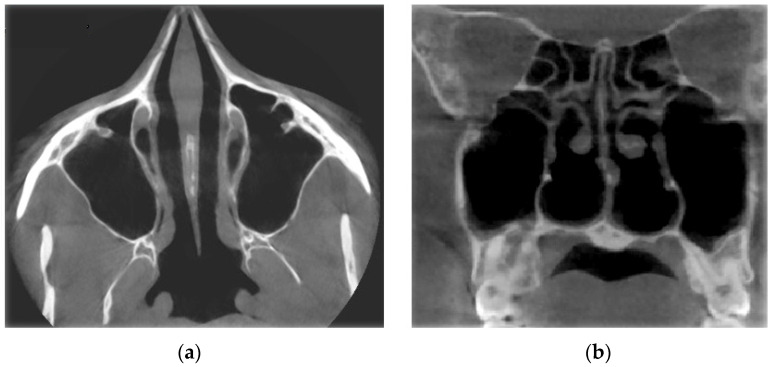
Computed tomography of a patient with empty nose syndrome after conchotomy—bilateral removal of the lower shells: (**a**) axial tomographic section; (**b**) multiplanar reconstruction in frontal projection.

Figure 17 represents tomographic data of a patient with empty nose syndrome after bilateral conchotomy. The removal of the lower shells and enlarged air space in the nasal cavity are clearly visualized in the axial (Figure 17a) and frontal (Figure 17b) projections.

**Figure 18:**
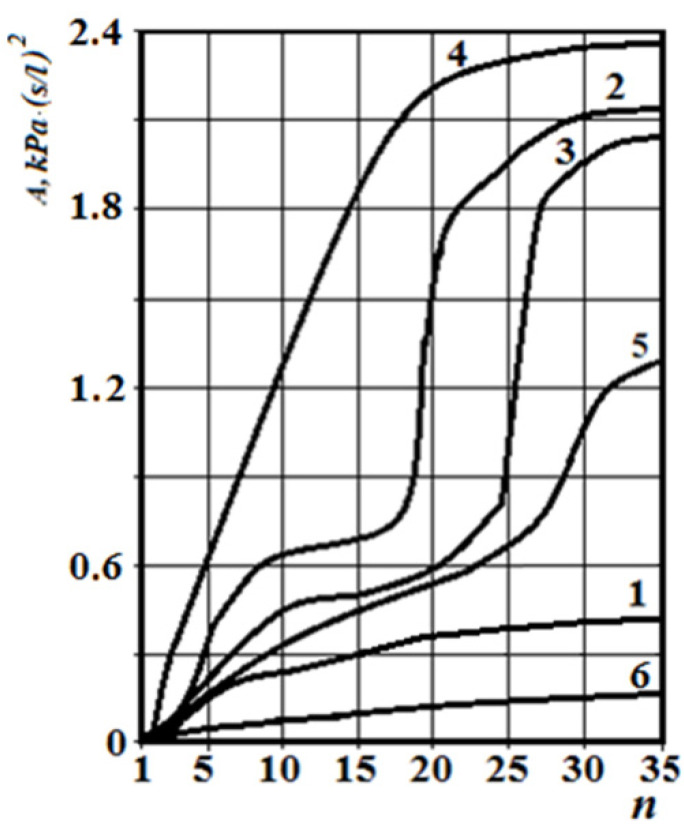
Graphs of the coefficient of aerodynamic nasal resistance growth by tomographic sections of the nasal cavity at different states of the nasal cavity: 1—conditional norm; 2—at curvature of a nasal partition in average departments; 3—at curvature of a nasal partition in back departments; 4—at chronic rhinosinusitis; 5—with adenoid vegetation; 6—with empty nose syndrome after conchotomy.

The corresponding graphs of the total coefficient of aerodynamic nasal resistance along the length of the nasal cavity at a distance between sections n equal to 2 mm are shown in Figure 18. The corresponding rhinomanometric graphs of the pressure drop in the nasopharynx from air flow are shown in figure resistance in contrast to Formula (2), in accordance with the quadratic regime of air flow (4), and it is calculated as:

(20) $$A=\frac{\Delta p}{Q^{2}}.$$

From Figure 18, it can be seen that at the conditional norm (curve 1), the coefficient of aerodynamic nasal resistance along the length of the nasal cavity increases sharply in the initial area (in the area of the nasal valve), and then there is a relatively smooth growth. When the nasal septum is curved (curves 2 and 3), there is a sharp increase in the coefficient of aerodynamic nasal drag in areas with cross-section numbers 17–25 (total length about 16 mm) and 25–28 (total length about 6 mm), respectively. The contribution of local aerodynamic drag, which is associated with the curvature of the nasal septum depending on the size of the area, is significantly larger in Figure 12 than with a more local offset. In chronic rhinosinusitis (curve 4) with generalized thickening of the mucous membrane of the nasal cavity, the coefficient of aerodynamic nasal resistance is greater than in other cases. During adenoid vegetations (curve 5) in the area (27–31, length about 8 mm), there is a rather sharp (but much smaller than in graphs 2 and 3) increase in the coefficient of aerodynamic nasal resistance, due to the location of aerodynamic resistance in a relatively wide distal part nasal cavity at the exit to the nasopharynx (see Figure 16). After conchotomy (curve 6), the aerodynamic resistance of the nasal cavity is significantly reduced and monotonically increases without abrupt changes and extremes, which is caused by a wide air channel.

**Figure 19:**
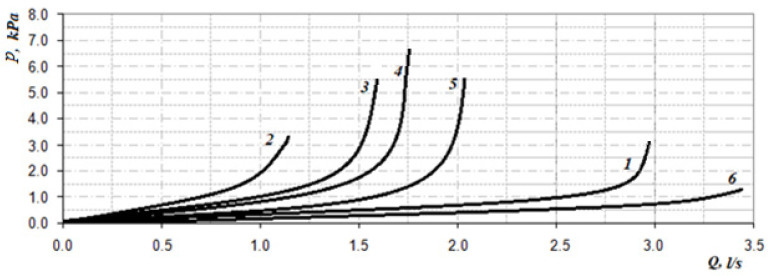
Graphs of the dependence of the pressure drop on the air flow according to the forced posterior active rhinomanometry at different states of the nasal cavity: 1–conditional norm; 2—at curvature of a nasal partition in average departments; 3—at curvature of a nasal partition in back departments; 4—at chronic rhinosinusitis; 5—with adenoid vegetation; 6—with empty nose syndrome after conchotomy.

The data of the functional study using posterior active rhinomanometry (see Figure 19) in the forced respiration actually fully correspond to the obtained analytical calculations in Figure 18. It can be concluded that the lowest air flow is at the curvature of the nasal septum (curves 2 and 3 on Figure 19), especially when the curvature of the nasal septum is in the middle section (curve 2 in Figure 13), which causes significant overlap of air flow by local resistance and subsequent turbulence of the flow for a considerable length. With generalized narrowing of the nasal canal due to chronic rhinosinusitis (curve 4 and Figure 14) there is also a fairly low air flow, but this is achieved due to a greater pressure drop, and as a consequence, the largest of these pathological coefficients of aerodynamic nasal drag with high pressure has a loss in length. Adenoid vegetations (curve 5 and Figure 16), also due to local narrowing in the nasopharynx, contribute to increased aerodynamic nasal resistance, but its increase is significantly (approximately twice) less than in the effects of chronic rhinosinusitis and curvature of the nasal septum.

**Figure 20:**
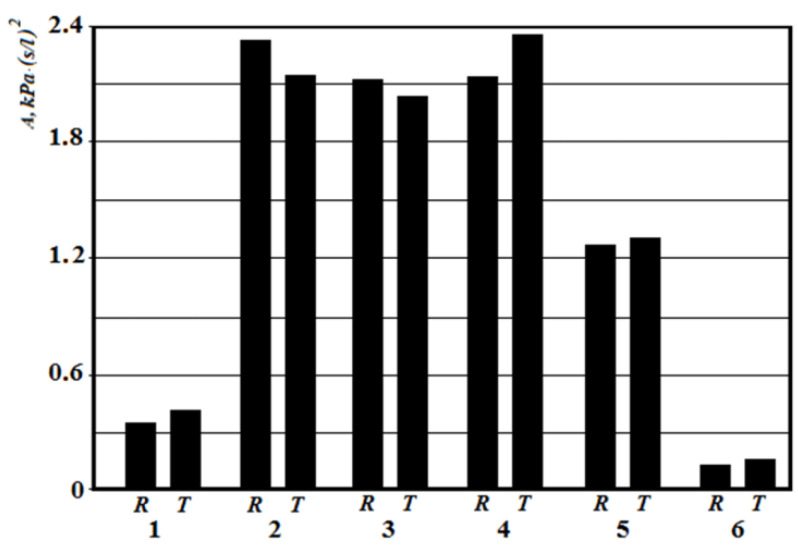
Graphs of the dependence of the pressure drop on the air flow according to the forced posterior active rhinomanometry at different states of the nasal cavity: 1—conditional norm; 2—at curvature of a nasal partition in average departments; 3—at curvature of a nasal partition in back departments; 4—at chronic rhinosinusitis; 5—with adenoid vegetation; 6—with empty nose syndrome after conchotomy.

At conchotomy (curve 6 and Figure 16), aerodynamic nasal resistance is significantly reduced, which causes high air flow at a small pressure drop. From the graphs in Figure 19, it can be clearly seen that according to the posterior active rhinomanometry during forced breathing, it is possible to clearly diagnose various disorders of the nasal cavity, both by air flow and by the coefficient of aerodynamic nasal resistance, which can be determined for both linear and quadratic modes of air flow by Formulas (2) and (20). The relative comparison of the coefficients of aerodynamic nasal resistance obtained experimentally by the results of rhinomanometry (Figure 19 and Formula (20)) and the data of the analytical model of air flow by tomographic sections according to Formulas (14)–(19) is shown in Figure 20. It should be borne in mind that in the laminar mode, there is a directly proportional relationship between the pressure drop and air flow, and the profile of air flow velocities in the sections is parabolic.

The highest values of the coefficients of aerodynamic nasal resistance were at the incisions of the nasal septum and rhinosinusitis. Even local resistance, as seen in the Figure 20, causes strong turbulence, which is not taken into account in the resistance model.

As shown in the review part of the work, the classic method of determining the aerodynamic characteristics of nasal breathing is rhinomanometry. Integral indicators of flow and pressure drop in different respiratory modes were analyzed in a large number of works [21]. However, from the point of view of the physiology of nasal breathing, it is necessary to consider the dynamic change of the air flow regime during forced breathing, which provides the maximum supply of oxygen to the lungs.

Knowledge of the air flow regime allows to determine the nature of the relationship between pressure drop and air flow during respiration, as well as to investigate the effect of air flow on the walls of the nasal cavity, given the distribution of velocities in the nasal passages.

From the diagrams in Figure 20, it can be seen that the total error between experimental rhinomanometric (R) and theoretical tomographic (T) is within 10%. The largest differences observed in the study of the coefficient of aerodynamic nasal resistance in the effects of rhinosinusitis (curve 4) and the conditional norm (curve 1) are due to the accumulation of errors in the representation of the characteristic dimensions of tomographic sections of the nasal cavity. At curvatures of a nasal partition (curves 2 and 3), the error which is caused by errors of analytical model of definition of local resistances is added to the general discrepancy. In this case, the error in the presence of local resistance in the rear parts has less effect on the error in determining the coefficient of aerodynamic nasal resistance, as the induced turbulence manifests itself at a shorter length of the air channel.

## 7. Possibilities of Testing Respiratory–Olfactory Disorders

The method of posterior active rhinomanometry can be used to test respiratory and olfactory disorders. This is possible by modifying the design of the device by placing the carrier odorivector with the subsequent determination of the energy characteristics of respiration. An illustration of a TNDA-PRH computer rhinomanometer with an olfactometric nozzle is shown in Figure 2. The olfactometric nozzle contains a container for an odorivector (for example, a hygroscopic cylindrical annular gasket impregnated with a solution of a specific odorous substance in the air path of the rhinomanometer). The patient performs breathing maneuvers with increasing intensity, which can be seen in the cyclogram of breathing, and fixes by pressing the button the time of sensitivity to the odorous substance. Next, the cyclograms of the pressure drop Δ*p*(*t*) and air flow *Q*(*t*) and the calculation of the pneumatic power *N*(*t*) of the breathing cyclogram are performed according to Formula (21):

(21) N(t)=Δp(t)⋅Q(t).

**Figure 21:**
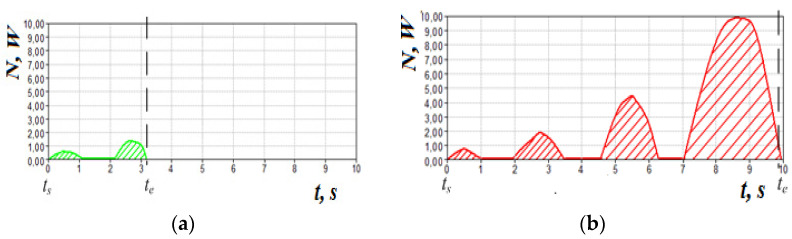
Cyclograms of pneumatic power at nasal breath: (**a**)—at conditional norm; (**b**)—at disturbance of olfactory sensitivity owing to rhinosinusitis.

The corresponding graphs of pneumatic power during normal breathing and in violation of olfactory sensitivity (the dashed line indicated the time of occurrence of sensitivity to the odor vector) are shown in Figure 21.

Determination of the energy *E* of respiration, which characterizes the colorimetric costs of respiration when the sensitivity to the odor vector is performed by integrating the data of the cyclogram of the pneumatic power of respiration (21) by the formula:

(22) $$E=\int_{t_{s}}^{t_{e}}N\left(t\right)dt,$$

where *t_s_*—start time of the study, as a rule, is taken equal to 0;

*t_e_*—time of appearance of sensitivity to the odor vector.

Integration is performed numerically by the trapezoidal method.

The classification of the degree of disturbance of odor perception was developed experimentally on the basis of the conducted research:

*E* ≤ 2 J—conditionally normal sense of smell;

2 < *E* ≤ 8 J—the average degree of dysosmia;

8 < *E* ≤ 16 J—severe dysosmia;

*E* > 16 J—almost complete dysosmia.

To study olfactory sensitivity, three olfactory substances of different receptor action are used: a solution of valerian with a concentration of 0.05%, which is due to the nerve n. olfactorius, 0.04% acetic acid, due to n. trigeminus and ammonia 0.004%, which is due to n. glossopharyngeus. In addition, for testing patients who have undergone COVID-19 with persistent olfactory loss, it is advisable to use the method of respiratory rehabilitation, which is based on determining the maximum olfactory sensitivity to household odorants (coffee, garlic, essential oils and some others) and stimulation training by inhalation of odor with high perception. The threshold of olfactory sensitivity can be determined in an automated mode by analyzing the cycles of respiration (Figure 21).

Thus, due to the placement of the odorant in the airway of the rhinomanometer, as well as procedures for determining the energy characteristics of respiration, it was possible to link respiratory parameters with olfactory function, which in turn allows for effective respiratory–olfactory testing of disorders at the evidentiary level.

**Figure 22:**
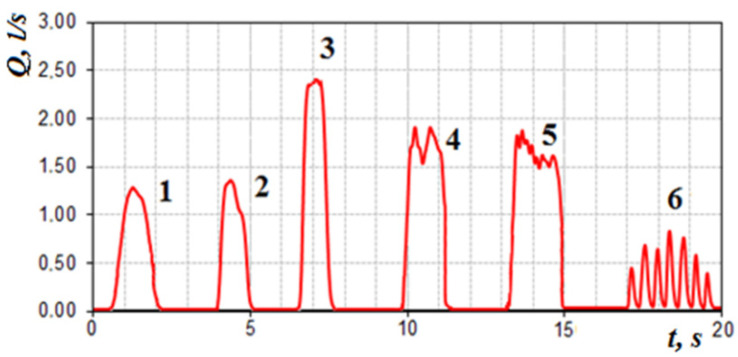
Options for respiratory cycles: 1, 2—calm breathing (normal); 3—forced breathing (rigidity of the nasal valve); 4, 5—forced breathing—stepped breathing (normally functioning mobility of the nasal valve); 6—stepped breath—“sniffing”.

Studies of rhinomanometry data in dynamic mode (with visualization of respiratory cyclograms) open new possibilities in the analysis and interpretation of the results of nasal breathing testing [8,64,65,66]. Thus, in Figure 22, the changes in the air flow are typical cycles of breathing with calm breathing in the norm (1 and 2, respectively).

In the forced mode of breath, it is possible to see the rigid nasal valve in Figure 22 (3) and the case of a nasal valve with normal functional mobility, which restricts the flow of air with a characteristic truncated vertex on the cyclogram (4 and 5, respectively), as well as stepped breath (6)—short “suction” of air, which can be described as a kind of “sniffing”, illustrated by the high-frequency beating of the signal on the air flow diagram.

**Figure 23:**
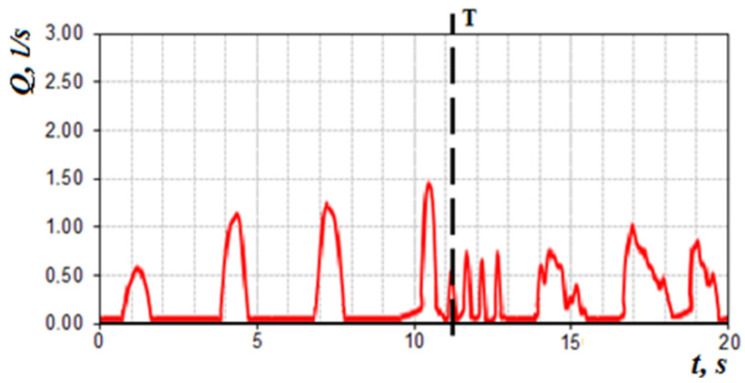
Cyclogram of air flow during nasal breathing (T—threshold of sensation).

When the odor vector is normal in the subject, being near the threshold of sensation (with increasing respiration rate) arbitrarily briefly increases respiration and at the threshold of sensation respiratory cycles (after the 4th respiratory cycle) in Figure 23 turns into “sniffing”, which promotes deeper penetration of air into the olfactory region and odor recognition. This moment of time can be characterized as the onset of the sensation threshold *T* odor vector (Figure 23).

## 8. Analysis of Errors in Rhinomanometric Measurements

**Figure 24:**
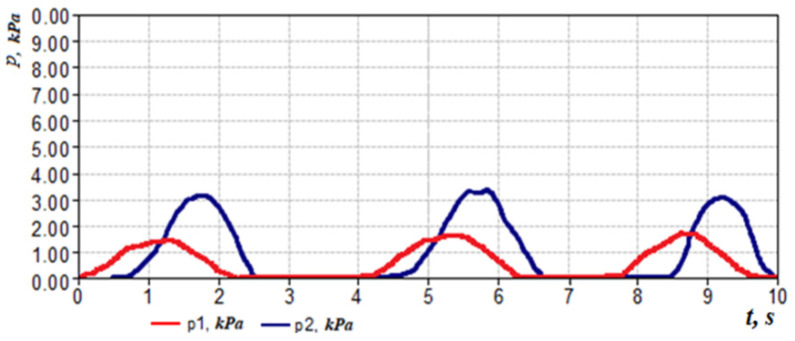
Illustration of registration of incorrect breathing maneuvers in the presence of a large amount of secretion in the nasal cavity.

The posterior active rhinomanometry method has its own characteristics and limitations, as well as possible errors and errors in measurements. Let us consider some of them. With an increased content of secretion in the nasal cavity, there is a significant out-of-phase between the pressure and flow signals in the respiratory cycle (see Figure 24, where a significant, almost in a quarter of a period, phase difference between the signals on the flow and pressure transducers is visualized), which, in turn, complicates both automatic and interactive definition of the existing measured values. Accordingly, such patients are shown to evacuate the contents of the nasal cavity before the study.

**Figure 25:**
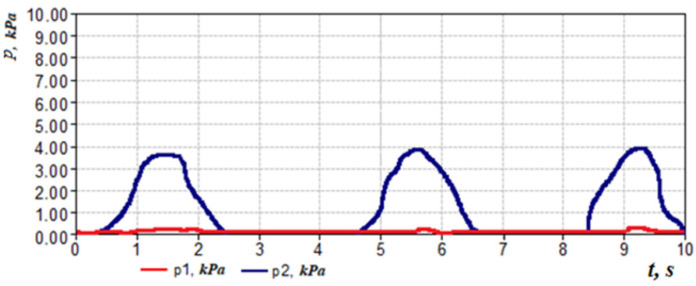
Illustration of registration of incorrect breathing maneuvers when the mask does not fit tightly to the patient’s face.

Loose (leaky) fit of the mask to the patient’s face leads to a significant decrease in the air flow rate (Figure 25, pressure transducer *p*_1_ shows pressure values close to zero).

In this case, it is necessary to select the shape and size of the mask, which ensures complete tightness of the connection in the place of its adhesion to the patient’s face. It is recommended to choose a mask with the smallest perimeter of fit to the face to make it easier to track possible cases of depressurization of the submask space.

**Figure 26:**
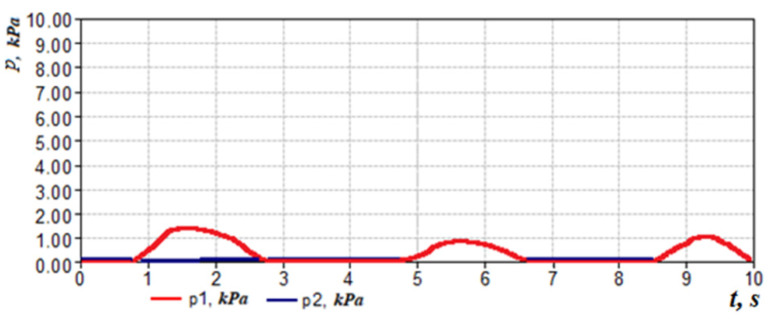
Illustration of registration of incorrect breathing maneuvers when the mouthpiece of apparatus is insufficiently gripped.

Insufficiently tight grip of the mouthpiece, leading to air leakage between it and the patient’s lips, or opening the mouth-air leakage from the corners of the mouth during posterior rhinomanometry, significantly reduces the pressure drop across the nasal cavity (see Figure 26, the *p*_2_ pressure transducer shows values close to zero pressure). It is necessary to provide a thin mouthpiece and a corresponding tight elastic obturator.

Closing the mouthpiece outlet with the tongue in the oral cavity during posterior rhinomanometry or strong compression of the mouthpiece with teeth or lips (with excessive flexibility) leads to a distortion of the *p*_2_ pressure drop in the nasal cavity (as a rule, towards a decrease in the indicator). It is necessary to provide a rigid mouthpiece and visually monitor in real time the process of rhinomanometric measurements.

**Figure 27:**
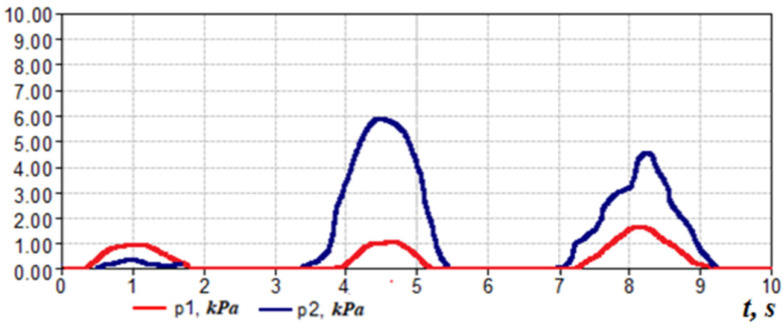
Illustration of registration of incorrect breathing maneuvers in case of perspiration in the oropharynx.

With the forced rhinomanometry, an improperly organized breathing maneuver will not lead to the development of high pneumatic power. It is expedient to recommend that the patient, while breathing, only forces the inhalation cycle as much as possible, and makes the exhalation smoother. Accordingly, in the event of a cough (third cycle in Figure 27) or other involuntary actions at the time of performing breathing maneuvers, repeated measurements should be taken. To determine the potential capabilities of the nasal cavity associated with the architectonics of osteochondral structures, it is advisable to perform forced posterior rhinomanometry with the preliminary introduction of vasoconstrictor drugs into the nasal cavity.

According to the discriminant analysis based on the examination of 286 patients with nasal breathing disorders and 60 people in the control group by three methods: endoscopy, computed tomography and posterior active rhinomanometry, the diagnostic significance of each method was established (for a specific sample of patients). The normalized Euclidean distance *δ* [31] was calculated to control between the norm and the violation of nasal breathing of each patient by each method, respectively. The probability of a diagnostic error was calculated, taking into account the integral of the Laplace probability by the formula

*P*_er_ = 1 − *Φ*(*δ*/2). (23)

**Figure 28:**
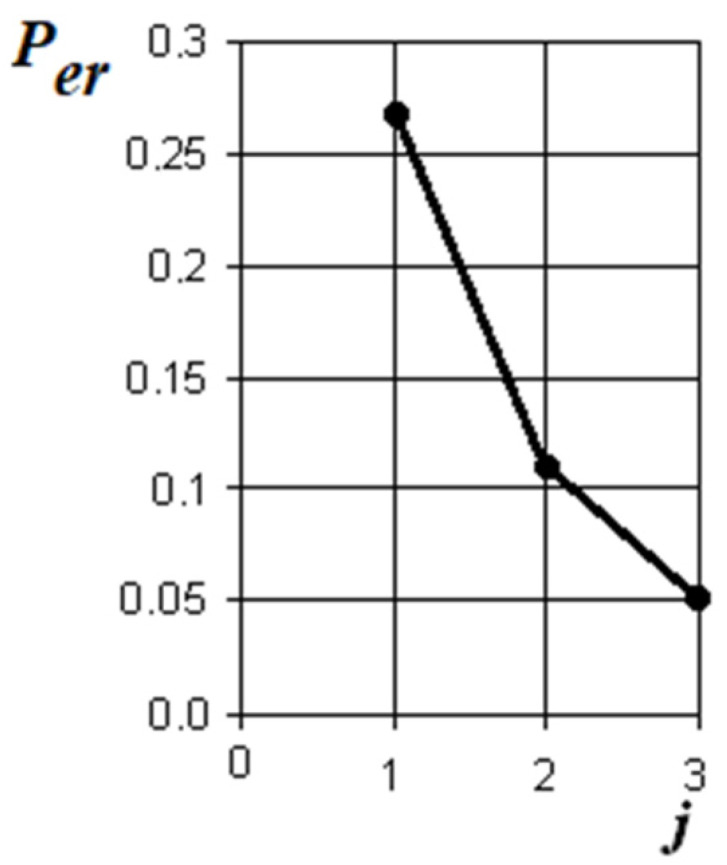
A graph of the decrease in the error in making a diagnostic decision when comparing a violation of nasal breathing with a conditional norm when adding data from various diagnostic methods: optical endoscopy, computed tomography, rhinomanometry (*j* = 3 is the dimension of the space of informative parameters).

The graph of the error in making a diagnostic decision is shown in Figure 28.

From the analysis of the graph in Figure 28, it is obvious that, according to the visual assessment of the state of the nasal cavity by endoscopy, the probability of an error in detecting nasal breathing disorders is 0.27 (the normalized Euclidean distance is 1.82). Taking into account the addition of computed tomography data to the discrimination model, the diagnostic error decreases to 0.11, which corresponds to the total normalized Euclidean distance of 3.19. When rhinomanometry data are added to the model, the total normalized Euclidean distance increases to 3.96, and the probability of making a diagnostic decision, respectively, decreases to 0.05. This allows to conclude that tomographic studies can significantly clarify the data of visual endoscopic examination of the nasal cavity. Rhinomanometric data make it possible to supplement the results of functional tests with information about changes in the architectonics of the nasal cavity by assessing the effect of anatomical structures on nasal aerodynamics and further reduce the likelihood of errors in diagnostic decisions when detecting nasal breathing disorders.

## 9. Conclusions

The analysis of the preliminary clinical trials of the TNDA computer rhinomanometer made it possible to identify some features of the operation of the means for the functional diagnosis of nasal breathing disorders and to develop the corresponding practical recommendations given below.

The method of posterior active rhinomanometry with forced breathing makes it possible to clearly differentiate various disorders of nasal breathing, both contributing to an increase in aerodynamic nasal resistance (curvature of the nasal septum, swelling of the mucous membrane with rhinosinusitis and polyposis processes) and a decrease in nasal resistance, for example, with conchotomy. In addition, it is the method of posterior active rhinomanometry that makes it possible to assess the contribution of epipharyngeal structures to nasal resistance by measuring the pressure drop in the nasopharynx from the oral cavity, which is important, for example, when assessing adenoid vegetations. The comparability of data on the coefficient of aerodynamic nasal resistance based on the results of posterior active rhinomanometry and the air flow model based on tomographic data is about 10%. Errors are explained by errors in the method for calculating local resistances and inconsistencies in the representation of channels of complex shape using the equivalent diameter. At the same time, only the combination of the results of functional rhinomanometric studies and topographic and anatomical data of computed tomography makes it possible to achieve correct anatomical and physiological interpretation when testing nasal breathing disorders.

According to the data of the discriminate analysis, it was shown that the probability of an error in detecting nasal breathing disorders when adding survey data by the method of posterior active rhinomanometry decreased from 0.11 to 0.05, which makes it possible to increase the reliability in making diagnostic decisions by evaluating functional information about nasal aerodynamics. When the rhinomanometry method is added to the examination of patients, it is additionally reduced by 0.06 to 0.05, which makes it possible to increase the reliability of detecting nasal breathing disorders in general.

It is advisable, according to the data of dynamic rhinomanometry, to assess the functioning of the nasal valve by the shape of the air flow rate signals during forced breathing and the structures of the soft palate by the residual nasopharyngeal pressure drop. It is imperative to take into account not only the maximum coefficient of aerodynamic nose drag, but also the values of the pressure drop and air flow rate in the area of transition to the turbulent quadratic flow regime.

From the point of view of the physiology of nasal breathing, it is necessary to consider the dynamic change of the air flow mode during the forced breathing, which provides the maximum supply of oxygen to the lungs. When planning functional rhinosurgical operations, it is necessary to apply the calculation method using computed tomography, which makes it possible to predict the functional result of surgery. When carrying out rhinomanometric measurements, as with any method of functional diagnostics, it is necessary to methodologically and correctly follow the examination protocol and monitor the correctness of the data obtained to exclude gross blunders. It is also necessary to indicate and take into account, in the protocol for the study of patients by the method of rhinomanometry, the air flow modes and the boundary areas of the change in the air flow modes.

For additional rear active rhinomanometry, it is possible to carry out testing of nasal damage by means of assessing the energy parameters of the nasal dysfunction in case of different odor vectors, which are used in the case of odor-metastatic rhinomanometry. It is especially important in case of sensitiveness to by-beat odorants and the conduct of mental training during the period of rehabilitation after the postponed COVID-19. The prospect of robots is taking into consideration infusion of characteristics of the surface of the mucous membrane on the nasal aerodynamics and the development of methods for monitoring the dichotomy for taking into consideration nasal cycle in case of advanced pathological camps.

## Figures and Tables

**Table 1:** Energy criterion of nasal breathing efficiency.

Energy Efficiency	$\mathrm{The}\;\mathrm{Coefficient}\;\mathrm{of}\;\mathrm{the}\;\mathrm{Main}\;\mathrm{Mode}\;\mathrm{of}\;\mathrm{Air}\;\mathrm{Flow}\;k_{T}$
Low	$k_{T}<30\%$
Average	$30\%\leq k_{T}\leq 60\%$
High	$k_{T}>60\%$

## Data Availability

The data were taken from the database of patients of the regional clinical hospital, the department of Otorhinolaryngology (Head and Neck Surgery), which is the clinical base for the department of Otorhinolaryngology of the Kharkov National Medical University.
